# Short-term morphological and functional outcomes of intravitreal faricimab for chorioretinal disorders: a real-world study in a Chinese population

**DOI:** 10.3389/fmed.2026.1909782

**Published:** 2026-08-20

**Authors:** Enzhong Jin, Xiaoying Zhu, Shijie Li, Mingwei Zhao, Heng Miao, Tong Qian

**Affiliations:** 1 Department of Ophthalmology, Peking University People's Hospital, Beijing, China; 2 Beijing Key Laboratory of Ocular Disease and Optometry Science, Peking University People's Hospital, Beijing, China; 3 Department of Ophthalmology, Beijing Yanhua Hospital, Beijing, China

**Keywords:** anti-vascular endothelial growth factor (anti-VEGF), chorioretinal disorders, faricimab, real-world study, short-term outcomes

## Abstract

**Objective:**

To evaluate the real-world efficacy and safety of the bispecific antibody faricimab in Chinese patients with neocascular age-related macular degeneration (nAMD), diabetic macular edema (DME), and macular edema secondary to retinal vascular occlusion (RVO-ME).

**Methods:**

This retrospective, single-center study included 63 patients (74 eyes: 47 nAMD, 19 DME, 8 RVO-ME); no subjects dropped out. Of the 74 eyes, 37.8% were treatment-naïve and 62.2% were treatment-experienced. All eyes received intravitreal faricimab 6 mg in a three-dose loading regimen followed by pro re nata (PRN) retreatment. Best-corrected visual acuity (BCVA, ETDRS letters) and central macular thickness (CMT) were assessed at baseline, 1 month, 3 months, and final follow-up (mean follow-up, 23.9 ± 12.3 weeks). Subgroup analyses by prior treatment status were performed.

**Results:**

Faricimab was associated with anatomical and functional improvements. In nAMD, treatment-naïve patients (*n* = 17) showed significant BCVA improvement (62.52 ± 5.80 to 71.05 ± 15.68 letters, *P* = 0.037) and CMT reduction (477.86 ± 189.45 to 324.19 ± 137.62 μm, *P* = 0.001) at final follow-up. Previously-treated nAMD patients (*n* = 30) showed only a transient CMT reduction at 3 months (*P* = 0.022) without sustained visual improvement. After the first injection, intraretinal fluid resolved in 90% of nAMD eyes that had IRF at baseline. After three loading doses, complete resolution of subretinal fluid and pigment epithelial detachment (PED) occurred in 66.7% and 31.3% of eyes, respectively. In DME, treatment-naïve patients (*n* = 9) achieved early BCVA improvement at 1 month (*P* = 0.0273) and sustained benefit at final follow-up (*P* = 0.0180), whereas previously-treated patients (*n* = 10) showed delayed improvement at 3 months in both BCVA and CMT (*P* = 0.0117 and 0.0195). In RVO-ME, BCVA improved significantly at 3 months and final follow-up (*P* = 0.018 and 0.028), whereas CMT showed a nonsignificant decrease. No significant ocular or systemic adverse events were observed.

**Conclusion:**

In this short-term real-world study comprising three exploratory disease-specific cohorts of Chinese patients, faricimab was associated with favorable anatomical and functional outcomes and was well tolerated, with the most pronounced benefit in treatment-naïve nAMD eyes. Responses in DME and RVO-ME were more variable and appeared to be influenced by prior treatment history; these subgroup analyses were exploratory, and larger prospective studies are warranted.

## 1 Introduction

Vascular endothelial growth factor (VEGF) has long been established as a pivotal mediator in the pathogenesis of chorioretinal vascular disorders, including neovascular age-related macular degeneration (nAMD), diabetic macular edema (DME), and retinal vein occlusion (RVO) (1–4). This foundational insight paved the way for anti-vascular endothelial growth factor (anti-VEGF) therapies to become the cornerstone of first-line treatment over the subsequent two decades (5). Despite well-documented efficacy from numerous randomized controlled trials (RCTs) and real-world studies demonstrating that anti-VEGF therapy successfully manages chorioretinal disorders and improves both anatomical and functional outcomes, significant challenges persist (6–8). These include partial or complete non-response in a proportion of patients, coupled with the substantial treatment burden and adherence issues arising from the need for frequent intravitreal injections (9, 10). This situation is particularly evident in China, where factors such as treatment response rate, comorbidity burden, and treatment adherence may further impact the effectiveness of existing therapies (11).

More recently, the angiopoietin/Tie-2 (Ang/Tie-2) signaling pathway has emerged as another critical regulator of retinal vascular stability and inflammation, offering a complementary therapeutic target (12). This rationale led to the development of faricimab (6 mg/0.05 mL, Vabysmo™, Roche/Genentech, Switzerland), a novel bispecific monoclonal antibody designed for dual inhibition of VEGF-A and angiopoietin-2 (Ang-2) pathways, which has since been validated for clinical use (13, 14). The efficacy and safety profiles of intravitreal faricimab (IVF) injections have been robustly demonstrated in several pivotal phases II and III clinical trials across nAMD, DME, and macular edema secondary to RVO (RVO-ME) (15–17). In these RCTs, faricimab achieved noninferiority to aflibercept in both best-corrected visual acuity (BCVA) gains and anatomical improvements; however, a geographical imbalance in the distribution of participants existed. Only 9% of participants in the TENAYA and LUCERNE trials (phase 3 trials of nAMD) were Asian, while only 10%−11% of participants in the YOSEMITE and RHINE trials (phase 3 trials of DME) were Asian (15, 16). Even in the BALATON and COMINO trials (phase 3 trials for RVO-ME), which included a higher proportion of Asian participants, the percentage ranged only between 23.0% and 30.8% (17). Consequently, the data pertaining to Asian populations remain insufficient, particularly among the Chinese population.

On the other hand, as with any novel therapeutic approach, a critical question remains: whether the outcomes observed in controlled trial settings translate to real-world clinical practice. Since its introduction three years ago, real-world evidence from regions such as Europe and North America has begun to accumulate (18, 19). Nonetheless, a significant evidence gap persists regarding its performance in the Chinese population, with a particular lack of studies featuring adequate sample sizes and longer-term follow-up. Existing reports from China remain limited in both scope and duration (20). To address this gap, reporting a real-world cohort of Chinese patients with chorioretinopathy who underwent IVF treatment is of great clinical value.

This study aims to generate real-world evidence for IVF treatment by reporting on a Chinese cohort that received the standard three-loading-dose regimen and was followed up for at least 3 months. We present both functional (BCVA) and anatomical outcomes at key time points: after the first injection, upon completion of the loading phase, and at the final follow-up, with subgroup analyses stratified by treatment history (treatment-naïve vs. previously-treated) for nAMD and DME. Our findings provide valuable insights into the clinical performance of faricimab in the management of nAMD, DME, and RVO-ME among Chinese patients, thereby helping to validate its real-world efficacy and safety.

## 2 Materials and methods

### 2.1 Ethical approval

This study was a single-center retrospective investigation conducted at the Department of Ophthalmology, Peking University People's Hospital (PKUPH) in China. The study protocol was reviewed and approved by the Ethical Review Committee of PKUPH and was conducted in accordance with the Declaration of Helsinki (2024PHB413-001). Written informed consent was obtained from each patient prior to enrollment.

Patients who received IVF treatment for nAMD, DME, and RVO-ME between April 1, 2024, and June 30, 2025, were included in this study. Both BRVO and CRVO were included in the RVO-ME group. And PCV, as an important phenotype, was included in the nAMD group. All enrolled patients were followed up for a minimum of 3 months after the initial IVF injection to ensure adequate assessment of treatment outcomes.

### 2.2 Study protocol

A systematic review of medical records of patients who underwent IVF treatment was performed to identify individuals diagnosed with nAMD, DME, or RVO-ME. Inclusion criteria for this study were patients aged 18 years or older with a minimum follow-up period of 3 months. A standardized three-dose loading regimen of faricimab following our center's routine practice was administered to all patients. The study population comprised both treatment-naïve individuals and those switched from prior anti-VEGF therapy, but a washout period of more than 4 weeks was required. Previously treated eyes were switched to faricimab primarily because of inadequate response to prior therapy. Exclusion criteria for this study included patients with a history of intravitreal corticosteroid therapy, significant media opacities or other ocular diseases that may interfere with the examination outcomes, or a history of prior anti-VEGF therapy within the past 4 weeks.

All patients underwent regular monthly follow-up assessments. Following completion of the three-dose loading therapy, the decision to administer retreatment was determined in accordance with the pro-re-nata (PRN) regimen based on follow-up findings. The PRN criteria included the presence of any fluid for nAMD eyes, or a central macular thickness (CMT) of ≥300 μm for DME and RVO-ME eyes (21, 22). All enrolled patients received IVF injections (6 mg in 0.05 mL) by the same retinal specialists (T.Q. and E.J.) following standard operating protocols in an operating room.

### 2.3 Outcome measures

All patients enrolled in our study underwent comprehensive ophthalmic examinations, including BCVA measured using the Early Treatment Diabetic Retinopathy Study (ETDRS) chart, fundus ophthalmoscopy, and optical coherence tomography (OCT). Changes in BCVA were assessed by comparing baseline values with the measurements at each visit during the follow-up period. The primary outcome was the change from baseline in BCVA and CMT. Secondary outcomes included the resolution of intraretinal fluid (IRF), subretinal fluid (SRF), and pigment epithelial detachment (PED) in nAMD patients, along with a comprehensive assessment of treatment safety.

Statistical analyses were performed on data from the following time points: (a) baseline, (b) 4 weeks after the initial IVF injection, (c) after the completion of three loading doses, and (d) the final visit. OCT imaging was conducted predominantly using one of two devices: the Cirrus HD-OCT (Carl Zeiss Meditec, Dublin, CA, USA) or the DREAM swept-source OCT system (Intalight, San Jose, CA). To ensure longitudinal consistency, all serial OCT scans for a given patient were acquired using the same device and a standardized imaging protocol throughout the study. OCT images were independently reviewed by two experienced retinal specialists (T.Q. and E.J.) Disagreements were resolved by consensus. However, image graders were not masked to clinical information or treatment status.

Subgroup analyses by treatment history (treatment-naïve vs. previously-treated) were performed for nAMD and DME; RVO-ME was analyzed as a single cohort because of the small sample size.

### 2.4 Statistical analysis

Baseline and demographic characteristics are summarized using descriptive statistics. The primary aim of this study is to evaluate the effectiveness of the reported real-world cohort data. The analysis is primarily based on quantitative outcomes, including changes in BCVA and CMT values, as well as the proportions of dichotomous variables (e.g., PED). Normality was assessed using the Shapiro–Wilk test. Continuous variables are presented as mean ± SD or median [IQR], as appropriate. Safety analysis consists of a comprehensive summary of relevant data. For nAMD, baseline-to-follow-up comparisons were performed using paired *t*-tests. For DME and RVO-ME, within-group changes were analyzed using the non-parametric tests (Friedman test followed by Wilcoxon signed-rank tests) because the data were non-normally distributed. Statistical analyses were performed using SPSS for Windows (version 21.0; IBM Corp., Armonk, NY, USA). Two-sided *P* < 0.05 was considered statistically significant.

## 3 Results

### 3.1 Demographical data

This study enrolled a total of 63 patients (74 eyes) with a mean age of 64.38 ± 13.57 years and a nearly equal sex distribution (34 females, 53.97%). The cohort comprised 43 patients (47 eyes) diagnosed with nAMD, 13 patients (19 eyes) with DME, and 7 patients (8 eyes) with RVO-ME. Among the enrolled eyes, 26 (35.14%) were treatment-naïve, while 48 (64.86%) eyes were switched from other anti-VEGF agents. Prior to switching to faricimab, this subgroup of patients had received a mean of 6.57 ± 4.68 injections of other anti-VEGF agents. At baseline, the mean BCVA for all affected eyes was 61.41 ± 14.23 ETDRS letters, and the mean CMT was 438.84 ± 188.44 μm. A total of 327 intravitreal faricimab injections were administered, corresponding to a mean of 4.42 ± 1.87 injections per eye over a mean follow-up of 23.93 ± 12.32 weeks, with a median of 20 weeks (range, 12–56 weeks), while 28 patients (44.4%) demonstrated a follow-up period exceeding 6 months (Table 1).

**Table 1:** Demographic characteristics of the study population.

Baseline variables	Values
Total number of patients (eyes)	63 (74)
Gender	
Male, *n* (%)	29 (46.03)f
Female, *n* (%)	34 (53.97)
Ages, y/o, mean (SD)	64.38 (13.57)
Treatment-naïve eyes, *n* (%)	26 (35.14)
Switching eyes, *n* (%)	48 (64.86)
Anti-VEGF injections prior to faricimab, mean (SD)	6.57 (4.68)
BCVA, ETDRS letters, mean (SD)	61.41 (14.23)
CMT, μm, mean (SD)	438.84 (188.44)
Diagnosis at baseline (eyes)	
nAMD, *n* (%)	47 (63.51)
DME, *n* (%)	19 (25.68)
RVO, *n* (%)	8 (10.81)

VEGF, vascular endothelial growth factor; BCVA, best-corrected visual acuity; ETDRS: early treatment diabetic retinopathy study; CMT, central macular thickness; nAMD, neovascular age-related macular degeneration; DME, diabetic macular edema; RVO, retinal vein occlusion.

### 3.2 nAMD group

Among 47 AMD eyes, 17 were treatment-naïve and 30 had received prior anti-VEGF therapy; Baseline demographic parameters were well-balanced between the treatment-naive and previously-treated subgroup. The mean age was 68.4 ± 6.2 years and 69.1 ± 5.8 years, respectively (*P* = 0.701). Males accounted for 58.8% (*n* = 10) of the treatment-naive and 60.0% (*n* = 18) of the previously-treated (P = 0.936). Baseline BCVA and CMT were comparable between the two subgroups. In the treatment-naïve subgroup (*n* = 17), ETDRS letters increased from 62.52 ± 5.80 at baseline to 66.20 ± 4.14 at 1 month (*P* = 0.019), 69.43 ± 11.84 at 3 months (*P* = 0.031), and 71.05 ± 15.68 at final follow-up (*P* = 0.037). CMT decreased from 477.86 ± 189.45 μm to 395.32 ± 143.98 μm, 347.41 ± 176.20 μm, and 324.19 ± 137.62 μm at the corresponding visits (*P* = 0.013, <0.001, and <0.001, respectively). In previously-treated eyes (*n* = 30), BCVA did not change significantly at any visit, whereas CMT decreased at 3 months only (434.46 ± 199.04 to 378.32 ± 128.59 μm, *P* = 0.022) (Table 2). The mean follow-up period for the AMD subgroup was 23.81 ± 12.74 weeks, with the treatment-naïve and previously-treated groups receiving 4.67 ± 1.63 and 4.30 ± 1.96 injections, respectively.

**Table 2:** Changes in the BCVA and CMT in nAMD population post faricimab injection.

Treatment-*n*aive group (*n* = 17)	Absolute value(Mean ±SD)	Mean change(95% CI)	*P*-value (vs. baseline)	Previously-*t*reated group (*n* = 30)	Absolute value (Mean ±SD)	Mean change (95% CI)	*P*-value (vs. baseline)
ETDRS score (letters)				ETDRS score (letters)			
Baseline	62.52 ± 5.80	—	—	Baseline	64.01 ± 11.22	—	—
1 month	66.20 ± 4.14	+3.69(0.70, 6.67)	0.0188^*^	1 month	65.60 ± 9.33	+1.59 (−2.17, 5.34)	0.394
3 months	69.43 ± 11.84	+6.91(0.73, 13.09)	0.0307^*^	3 months	68.04 ± 7.13	+4.03 (−0.08, 8.14)	0.0545
Final follow-up	71.05 ± 15.68	+8.53(0.59, 16.48)	0.0369^*^	Final follow-up	64.34 ± 14.48	+0.33 (−4.31, 4.97)	0.887
CMT thickness (μm)				CMT Thickness (μm)			
Baseline	477.86 ± 189.45	—	—	Baseline	434.46 ± 199.04	—	—
1 month	395.32 ± 143.98	−82.54 (−145.04, −20.04)	0.0129^*^	1 month	394.15 ± 135.35	−40.31 (−109.73, 29.11)	0.2446
3 months	347.41 ± 176.20	−130.46 (−194.62, −66.29)	0.0005^*^	3 months	378.32 ± 128.59	−56.14 (−103.49, −8.79)	0.0218^*^
Final follow-up	324.19 ± 137.62	−153.68 (−232.24, −75.11)	0.0008^*^	Final follow-up	422.86 ± 131.26	−11.60 (−88.77, 65.56)	0.7607

^*^P-value statistically significant. BCVA, best-corrected visual acuity; CMT, central macular thickness; nAMD, neovascular age-related macular *degeneration*; ETDRS, early treatment diabetic retinopathy study.

We also evaluated the resolution of fluid and pigment epithelial detachment (PED). The proportions of eyes with subretinal fluid (SRF), intraretinal fluid (IRF), and PED at baseline were 18/47 (38.3%), 10/47 (21.3%), and 32/47 (68.1%), respectively. These proportions decreased to 9/47 (19.1%), 1/47 (2.1%), and 28/47 (59.6%) at the 1-month visit, and further to 6/47 (12.8%), and 22/47 (46.8%) for SRF and PED, respectively, while the proportion of IRF was 3/47 (6.4%) after three loading doses.

### 3.3 DME group

In the eyes with DME (*n* = 19), 9 were treatment-naïve and 10 were previously-treated. The mean age was 54.3 ± 17.8 years for the treatment-naïve group and 62.5 ± 5.6 years for the previously-treated subgroup, showing no significant difference (*P* = 0.185). Similarly, the sex distribution did not differ significantly between the two subgroup (66.7% vs. 30.0% males, respectively; *P* = 0.179). In treatment-naïve eyes (*n* = 9), median BCVA improved from 58.86 [50.05, 73.91] letters at baseline to 69.95 [58.86, 73.91] at 1 month (*P* = 0.027) and 80.15 [77.25–80.15] at final follow-up (*P* = 0.018). Median CMT was 438.19 [372.40–462.00] μm at baseline, showed a significant reduction at the 1-month follow-up (303.00 [229.50 – 350.00] μm, *P* = 0.0039) and 3-month (329.26 [227.30, 347.20] μm, *P* =0.004), but no significant anatomical difference from baseline was noted at the final visits (*P* = 0.250). In previously-treated eyes (*n* = 10), BCVA improved significantly only at 3 months (57.57 [37.20, 65.10] to 71.93 [58.66 – 79.43] letters, *P* = 0.012), with a corresponding CMT change at 3 months (418.00 [357.50–454.00] to 364.92 [342.93, 401.48] μm, *P* = 0.020) (Table 3). Patients in the DME subgroup were followed for a mean of 24.00 ± 13.56 weeks, averaging 4.14 ± 2.04 injections in the treatment-naïve cohort and 4.43 ± 2.57 injections in the previously treated cohort.

**Table 3:** Changes in the BCVA and CMT in DME population post faricimab injection.

Treatment-naïve (*n* = 9)	Median [IQR]	*P*-value (vs. baseline)	Previously-treated (*n* = 10)	Median [IQR]	*P*-value (vs. baseline)
ETDRS score (letters)			ETDRS score (letters)		
Baseline	58.86 [50.05, 73.91]	-	Baseline	57.57 [37.20, 65.10]	-
1 month	69.95 [58.86,73.91]	0.0273^*^	1 month	67.53 [51.26, 69.95]	0.4413
3 months	73.91 [58.86, 73.91]	0.0742	3 months	71.93 [58.66, 79.43]	0.0117^*^
Final visit	80.15 [77.25, 80.15]	0.0180^*^	Final visit	71.93 [55.89,76.41]	0.2324
CMT thickness (μm)			CMT thickness (μm)		
Baseline	438.19 [372.40, 462.00]		Baseline	418.00 [357.50, 454.00]	-
1 month	303.00 [229.50, 350.00]	0.0039^*^	1 month	421.25 [409.67,445.45]	0.9219
3 months	329.26[227.30, 347.20]	0.004^*^	3 months	364.92 [342.93, 401.48]	0.0195^*^
Final follow-up	394.91 [321.40, 412.10]	0.2500	Final follow-up	373.15 [355.40, 400.05]	0.6953

^*^P-value statistically significant. BCVA, best-corrected visual acuity; CMT, central macular thickness; DME, diabetic macular edema; ETDRS, early treatment diabetic retinopathy study.

### 3.4 RVO-ME group

In eyes with RVO-ME (*n* = 8), all of the patients were previously-treated patients. The median ETDRS score improved from 51.33 [35.00–67.30] letters at baseline to 73.91 [65.10–75.47] at 3 months (*P* = 0.018), and 69.95 [68.74–73.91] at final follow-up (*P* = 0.028). CMT decreased from 471.08 [399.20–532.88] μm at baseline to 336.11 [317.20–378.00] μm at 1 month, 342.90 [314.30–365.35] μm at 3 months, and 338.55 [302.60–392.00] μm at final follow-up. Similarly, pairwise comparisons between baseline and subsequent follow-up points (1 month, 3 months, and final follow-up) showed no statistically significant differences (*P* > 0.05 for all), likely due to the small sample size (Table 4).

**Table 4:** Changes in the BCVA and CMT in RVO-ME population post faricimab injection.

Characteristics	Baseline	1 month	3 months	Final follow-up
ETDRS score (letters)	51.33 [35.00, 67.30]	65.10 [52.13, 66.31]	73.91 [65.10, 75.47]	69.95 [68.74, 73.91]
*P*-value vs. Baseline^a^	—	0.344	0.018^*^	0.028^*^
CMT thickness (μm)	471.08 [399.20, 532.88]	336.11 [317.20, 378.00]	342.90 [314.30, 365.35]	338.55 [302.60, 392.00]
*P*-value vs. Baseline^a^	—	0.161	0.093	0.093

^*^P-value statistically significant. ^a^Wilcoxon signed-rank test for comparison with baseline. Data are presented as Median [Interquartile Range]. BCVA, best-corrected visual acuity; CMT, central macular thickness; RVO, retinal vein occlusion; ETDRS, early treatment diabetic retinopathy study.

**Figure 1:**
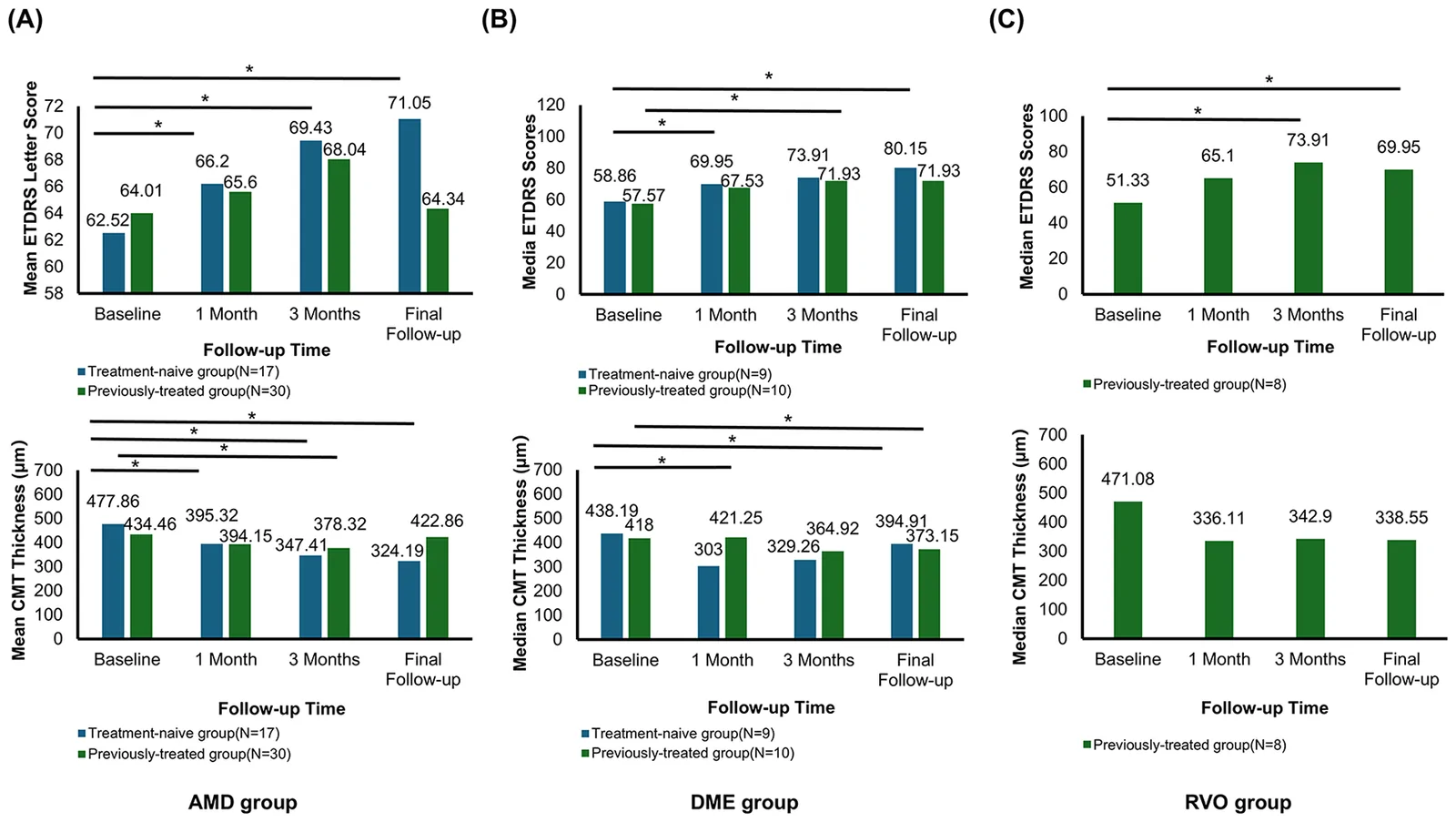
Best-corrected visual acuity (BCVA) and central macular thickness (CMT) changes during follow-up. Bar charts showing ETDRS scores (top row) and CMT (bottom row) for **(A)** AMD, **(B)** DME, and **(C)** RVO patients at baseline, 1 month, 3 months, and final follow-up. Blue bars represent treatment-naive patients; green bars represent previously-treated patients. *indicate statistically significant differences between the connected time points. AMD, age-related macular degeneration; DME, diabetic macular edema; RVO, retinal vein occlusion; ETDRS, early treatment diabetic retinopathy study.

The changes in BCVA and CMT for the three groups are shown in Figure 1.

### 3.5 Safety

During the entire follow-up period, no occurrences of elevated intraocular pressure were observed among the patients. The study population did not report any instances of endophthalmitis, vitreous hemorrhage, retinal tears, or treatment-related cataract exacerbation. More importantly, no serious systemic adverse events defined according to the APTC criteria (including nonfatal myocardial infarction, nonfatal stroke, and cardiovascular death) were reported in this study population.

## 4 Discussion

The present study evaluated the short-term real-world efficacy and safety of IVF in patients with nAMD, DME, and RVO-ME in a Chinese population. In this study, we demonstrated the efficacy of the 3 + PRN faricimab regimen for the treatment of chorioretinal disorders including nAMD, DME, and RVO-ME, in both treatment-naïve individuals and those switched from prior anti-VEGF therapy.

The therapeutic rationale for faricimab lies in its dual antagonism of VEGF-A and Ang-2 (14). This combined action not only suppresses neovascularization but also promotes vascular stabilization, a mechanistic advantage that may underlie the rapid fluid resolution and sustained anatomical improvements observed in clinical trials and real-world evidence, particularly in refractory cases (15–20). The present study findings demonstrated improvements in BCVA and CMT across all diagnostic groups, especially in treatment-naïve nAMD eyes, with a notable reduction in IRF and SRF across the entire nAMD cohort. DME showed a more heterogeneous response according to prior treatment history, and RVO showed clear functional improvement with a weaker anatomical signal. Faricimab was also well tolerated, with no significant ocular or systemic adverse events reported. These findings are broadly consistent with the disease-specific efficacy profiles reported in the pivotal faricimab trials and highlight the value of stratified analysis rather than pooled mean-based comparisons, particularly in skewed datasets with small subgroup sizes (15, 16).

In this real-world study, patients with nAMD constituted the largest subgroup. This can be attributed to the sequential inclusion of conditions in China's National Reimbursement Drug List (NRDL). Prior to faricimab's NRDL listing, the high out-of-pocket cost may have selected for a higher proportion of treatment-refractory nAMD patients switching from other therapies. Following reimbursement for nAMD and DME, CRVO-ME remained non-reimbursed during our enrollment period, explaining its lower representation. The mean follow-up was approximately 6 months. However, as the NRDL inclusion was recent at the time of analysis, only 44.4% of patients had a follow-up period exceeding 6 months, which constitutes a study limitation. Future prospective studies with larger samples and extended follow-up are warranted to generate more robust real-world evidence.

Among the nAMD cohort in this real-world study, treatment-naïve eyes derived the most pronounced short-term benefit from the intravitreal faricimab loading regimen, with both functional and anatomical gains sustained over follow-up. Previously-treated nAMD eyes showed a weaker and less durable response, which may reflect chronic structural damage, residual disease activity, a smaller reversible component or a ceiling effect after prior anti-VEGF exposure. In other words, once photoreceptor and outer retinal injury become established, anatomical drying may no longer translate into equally large functional gains. These data suggest the importance of earlier introduction of faricimab may be more likely to preserve vision in nAMD.

Notably, as a prevalent feature in nAMD that signifies disease activity and impacts prognosis, PED was present in 68.1% of eyes at baseline, a rate consistent with existing literature (23). Following faricimab therapy, a continuous reduction in PED incidence was observed, decreasing by approximately one-third after the completion of the loading doses. This provides real-world evidence supporting the modulating effect of faricimab on this persistent anatomical structure. Furthermore, analysis of fluid compartments revealed that faricimab induced rapid and substantial resolution. Intraretinal fluid (IRF) was nearly completely resolved after a single injection, while the prevalence of subretinal fluid (SRF) was significantly reduced and further declined to 12.8% following the loading regimen. These robust data collectively indicate that faricimab effectively promotes the absorption of exudative components in nAMD, leading to anatomical stabilization and corresponding visual benefit (15). The fluid resolution dynamics observed in our real-world nAMD cohort aligned closely with those reported in the pivotal TENAYA/LUCERNE phase III trials and mirrored the high fluid absorption rates documented by Yefung et al. (20) in refractory nAMD, collectively corroborating the treatment's effectiveness in clinical practice (15). Notably, the rates of complete pigment epithelial detachment (PED) regression (12.5% after the first loading dose and 31.25% after three doses) resembled the patterns observed in real-world studies such as that by Cancian et al. (24) suggesting that achieving significant PED regression in routine practice often requires an extended treatment course.

In patients with DME, treatment-naïve eyes achieved earlier improvement and sustained this benefit through the end of follow-up, whereas previously-treated eyes showed a delayed response at 3 months. The observed positive trends in BCVA improvement and CMT reduction, align with the established efficacy of faricimab demonstrated in large-scale trials and growing real-world evidence (16, 25). The delayed response in our previously-treated cohort may reflect chronic diabetic microvascular injury and retinal structural disorganization. In particular, disorganization of the retinal inner layers has been associated with poorer visual prognosis in DME, and broader anti-VEGF data suggest that baseline retinal integrity and chronicity modulate functional recovery (26, 27). Thus, the magnitude and timing of response in DME may depend not only on fluid resolution, but also on the extent of irreversible retinal damage. Therefore, our findings, while preliminary, contribute to the real-world profile of faricimab in DME.

In the RVO-ME subgroup, median BCVA improved significantly over time, but CMT did not reach statistical significance. This mismatch between functional and anatomical outcomes is not unusual in anti-VEGF-treated RVO, where visual recovery may precede complete thickness normalization (28, 29). In a small cohort, CMT is particularly vulnerable to limited statistical power, baseline heterogeneity, measurement variability and residual macular ischaemia. Therefore, the absence of a significant CMT change should not be interpreted as lack of clinical benefit, especially when visual acuity improved. Although the small cohort size underscores the need for confirmation in larger studies, the observed positive trajectory provides an encouraging signal of the therapeutic potential of faricimab in this indication.

Although this study provides early real-world evidence on faricimab for chorioretinal vascular diseases in a Chinese population, offering valuable insights for clinical practice in this demographic, several limitations merit consideration. First, the retrospective single-center design may introduce selection bias and limits external generalizability, and single-arm design inherently limits the ability to establish comparative efficacy against other agents or control groups, and confines the analysis to outcomes associated with faricimab monotherapy. Second, the relatively modest sample size may underpower the statistical analysis, especially for the RVO-ME subgroup, particularly for detecting more nuanced treatment effects. The subgroup analyses, particularly those involving DME and RVO-ME subgroup, were limited by small sample sizes and should therefore be considered exploratory and hypothesis-generating, and larger cohorts are needed to validate and strengthen these findings. Third, patients in the present study received traditional 3-loading-dose followed by PRN follow-up, which differs from both standard drug instructions and the modern 4-loading-dose plus treat-and-extend (T&E) regimen widely used currently, which may not be fully representative. Future prospective studies with expanded sample sizes are warranted to address these gaps and enable more granular investigations into specific patient subgroups. Finally, several statistical limitations should be acknowledged. Multiple pairwise comparisons were performed across different follow-up time points (1, 3 month, and final follow-up time) for both BCVA and CST without formal multiple testing corrections. This approach inevitably inflates the cumulative risk of Type I errors (false positives). Consequently, some of the borderline significant findings should be interpreted with caution and treated as hypothesis-generating rather than definitive. Future confirmatory studies with larger sample sizes are warranted to validate these longitudinal trajectories.

Faricimab, with its dual-pathway inhibition of VEGF-A and Ang-2, represents a paradigm shift in managing chorioretinal vascular diseases. Global evidence has corroborated its efficacy, safety, and potential to extend treatment intervals, thereby reducing patient burden (12–20). While early data from Asian populations are promising, robust real-world evidence specific to clinical practice in China remains nascent (22). This study provides preliminary real-world observations regarding the treatment-associated anatomical and functional changes following faricimab therapy across nAMD, DME, and RVO-ME in a Chinese cohort. To fully establish its long-term value and optimize treatment protocols, future prospective studies with larger sample sizes and extended follow-up are warranted.

## 5 Conclusion

In conclusion, this short-term real-world cohort study demonstrated that intravitreal faricimab, administered as a three-dose loading regimen followed by PRN retreatment, was associated with both anatomical and functional improvements and was well tolerated. The most pronounced benefits were observed in treatment-naïve nAMD eyes. Responses in DME and RVO-ME were more heterogeneous, particularly in treatment-experienced eyes. Larger prospective studies are needed to confirm these findings, and further research is warranted to evaluate the comparative efficacy and durability of faricimab vs. conventional therapies in Chinese populations to inform future treatment strategies.

## Data Availability

The original contributions presented in the study are included in the article/supplementary material, further inquiries can be directed to the corresponding authors.
